# Antimicrobial prescribing for urinary tract infections in patients undergoing total hip or knee arthroplasty (THA/TKA)

**DOI:** 10.1186/2047-2994-4-S1-P220

**Published:** 2015-06-16

**Authors:** S Bailin, N Noiseux, J Pottinger, B Johannsson, A Haleem, S Johnson, L Herwaldt

**Affiliations:** 1 University of Iowa College of Medicine, Iowa City, USA; 2 Orthopaedics, University of Iowa College of Medicine, Iowa City, USA; 3 Clinical Quality, Safety, and Process Improvement, University of Iowa Hospitals and Clinics, Iowa City, USA; 4 Internal Medicine, Landspitali University Hospital, Reykjavik, Iceland; 5 Internal Medicine, University of Iowa College of Medicine, Iowa City, USA; 6 Pharmaceetical Care, University of Iowa Hospitals and Clinics, Iowa City, USA

## Introduction

Patients undergoing THA/TKA were screened pre- & postoperatively with urinalysis (UA) including urine dipstick & microscopy. We hypothesized that: 1) many patients without evidence of urinary tract infection (UTI) receive antibiotics if clinicians base treatment on UA results alone; 2) a protocol for screening patients for UTI would decrease treatment for UTI without increasing surgical site infection (SSI) rates.

## Objectives

To identify determinants of treatment for UTI among patients undergoing THA or TKA; to assess the effect of implementing a protocol for screening patients for UTI before THA/TKA on antimicrobial use and SSI rates.

## Methods

We conducted a retrospective cohort study of 200 consecutive patients undergoing THA/TKA from 2/21 - 6/30/2011 & a prospective cohort study of 50 patients undergoing these procedures from 5/21 - 7/17/2012 to identify factors influencing treatment for UTIs. We conducted a before-after study to assess the outcome of implementing a screening protocol.

## Results

The strongest determinants of pre- or postoperative treatment for UTI were positive leukocyte esterase (LE; P < 0.0001; P < 0.0001) and urine white blood cell count > 5 (P = 0.01; P = 0.01). At least 59.7% of patients treated did not have clinical evidence of UTI. The screening protocol was revised such that all patients with a positive LE or nitrite test have urine cultures. Patients were treated for UTIs if the cultures grow > 100,000 CFU of 1 organism. Subsequently, the number of patients receiving antimicrobial treatment for presumed UTI decreased 80.2%; the SSI rate did not increase.

## Conclusion

A new protocol for diagnosis and treatment of UTIs was associated with a significant decrease in treatment for presumed UTI but the incidence of SSI did not increase. These results suggest that most patients with positive LE do not need treatment for UTI before THA/TKA.

## Disclosure of interest

None declared.

